# Panmicrobial Oligonucleotide Array for Diagnosis of Infectious Diseases

**DOI:** 10.3201/eid1301.060837

**Published:** 2007-01

**Authors:** Gustavo Palacios, Phenix-Lan Quan, Omar J. Jabado, Sean Conlan, David L. Hirschberg, Yang Liu, Junhui Zhai, Neil Renwick, Jeffrey Hui, Hedi Hegyi, Allen Grolla, James E. Strong, Jonathan S. Towner, Thomas W. Geisbert, Peter B. Jahrling, Cornelia Büchen-Osmond, Heinz Ellerbrok, Maria Paz Sanchez-Seco, Yves Lussier, Pierre Formenty, Stuart T. Nichol, Heinz Feldmann, Thomas Briese, W. Ian Lipkin

**Affiliations:** * Columbia University, New York, New York, USA; † Stanford University, Stanford, California, USA; ‡ University of Chicago, Chicago, Illinois, USA; § Institute of Enzymology, Budapest, Hungary; ¶ Public Health Agency of Canada, Winnipeg, Manitoba, Canada; # Centers for Disease Control and Prevention, Atlanta, Georgia, USA; ** US Army Medical Research Institute of Infectious Diseases, Fort Detrick, Frederick, Maryland, USA; †† National Institutes of Health Integrated Research Facility, Fort Detrick, Frederick, Maryland, USA; ‡‡ Robert Koch Institut, Berlin, Germany; §§ Instituto de Salud Carlos III, Madrid, Spain; ¶¶ World Health Organization, Geneva, Switzerland; ## University of Manitoba, Winnipeg, Manitoba, Canada; 1 These authors contributed equally to this study.

**Keywords:** Malaria, viral hemorrhagic fever, differential diagnosis, oligonucleotide microarrays, microarray analytical devices, communicable diseases, emerging communicable disease control, research

## Abstract

To facilitate rapid, unbiased, differential diagnosis of infectious diseases, we designed GreeneChipPm, a panmicrobial microarray comprising 29,455 sixty-mer oligonucleotide probes for vertebrate viruses, bacteria, fungi, and parasites. Methods for nucleic acid preparation, random primed PCR amplification, and labeling were optimized to allow the sensitivity required for application with nucleic acid extracted from clinical materials and cultured isolates. Analysis of nasopharyngeal aspirates, blood, urine, and tissue from persons with various infectious diseases confirmed the presence of viruses and bacteria identified by other methods, and implicated *Plasmodium falciparum* in an unexplained fatal case of hemorrhagic feverlike disease during the Marburg hemorrhagic fever outbreak in Angola in 2004–2005.

Rapid differential diagnosis of infectious diseases is increasingly important as novel pathogens emerge in new contexts and treatment strategies are beginning to be tailored to specific infectious agents. Because clinical syndromes are rarely specific for single pathogens, unbiased multiplex assays are essential. Methods for direct molecular detection of microbial pathogens in clinical specimens are rapid, sensitive, and may succeed when fastidious requirements for agent replication or the need for high-level biocontainment confound cultivation.

We have adopted a staged strategy for molecular pathogen surveillance and discovery. In the first stage we use MassTag PCR, a PCR platform wherein discrete mass tags rather than fluorescent dyes serve as reporters. This method, which allows simultaneous detection of >20 different pathogens with high sensitivity, has proven useful for differential diagnoses of respiratory disease and viral hemorrhagic fevers (*1*–*3*). However, it is not sufficient when larger numbers of known pathogens must be considered, when new but related pathogens are anticipated, or when sequence divergence might impair binding of PCR primers. Thus, to address the challenge of more highly multiplexed differential diagnoses, we established an oligonucleotide microarray platform.

Microarrays have potential to provide a platform for highly multiplexed differential diagnosis of infectious diseases (*4*,*5*). The number of potential features per microarray far exceeds those of any other known technology; hundreds of thousands of features can be printed on 70-mm × 20-mm slides. Furthermore, sequence probes of >70 nt are not uncommon. Thus, microbes can be detected when melting temperatures are high enough to allow hybridization, despite a lack of precise complementarity between probe and target. Lastly, microbial and host gene targets can be incorporated, which provides an opportunity to detect microbes and assess host responses for signatures consistent with various classes of infectious agents. Despite these advantages, microbial arrays have not been widely used with clinical materials because of limited sensitivity. The primary service of microbial arrays has been characterization of agents propagated to high titer in vitro (*6*).

We report establishment of a microarray platform for pathogen surveillance and discovery, the GreeneChip system. Its key features include a comprehensive microbial sequence database for probe design and protocols for sample preparation, amplification, labeling, hybridization, and analysis. The system has been optimized with cultured viral isolates; tested with blood, respiratory, urine, and tissue samples containing bacterial and viral pathogens; and applied in an outbreak investigation when other methods failed to implicate a microorganism in fatal hemorrhagic fever case.

## Methods

### Pathogen Database

A vertebrate viral sequence database (GreeneVrdB) was established by integrating the database of the International Committee on Taxonomy of Viruses (ICTVdB, http://phene.cpmc.columbia.edu), a database that describes viruses at the levels of order, family, genus, and species, and the sequence database of the National Center for Biotechnology Information (NCBI, http://www.ncbi.nih.gov). Functionally related sequences were clustered by using the protein families (Pfam, http://pfam.wustl.edu) database of alignments (*7*). Most viral protein coding sequences in the NCBI database (84%) were represented in the Pfam database; the remainder were mapped by using pairwise BLAST alignments (*8*). The rRNA sequences of fungi, bacteria, and parasites obtained from the Ribosomal Database Project (RDP, http://rdp.cme.msu.edu) or the NCBI database were added to create a panmicrobial database (GreenePmdB). The GreenePmdB comprises the 228,638 viral sequences of the GreeneVrdB that represent complete and partial viral genomes: 41,790 bacterial 16S rRNAs, 4,109 fungal 18S rRNAs, and 2,626 18S parasitic rRNAs. These sequences represent all recognized 1,710 vertebrate virus species and 135 bacterial, 73 fungal, and 63 parasite genera.

### GreeneChip Design and Fabrication

Viral probes were designed to represent a minimum of 3 distinct genomic target regions for every family or genus of vertebrate virus in the ICTVdB. When possible, we chose highly conserved regions within a coding sequence for an enzyme such as a polymerase and 2 other regions that corresponded to more variable structural proteins. We thought that RNAs that encode structural proteins may be present at higher levels than those that encode proteins needed only in catalytic amounts and that use of probes representing noncontiguous sites along the genome might allow detection of naturally occurring or intentionally created chimeric viruses.

Any diagnostic tool based on nucleic acid hybridization is necessarily dependent on the extent to which probes are complementary to their targets. Although sequence databases are increasingly comprehensive, it is unlikely that more than a fraction of the existing microbial sequence space has been explored. Our intent in implementing GreeneChip was to have the potential to identify known and related agents for which precise sequence information was not available. To assess the extent to which a given probe sequence can hybridize to a nonmatching but related sequence, we analyzed synthetic mismatch controls. Whereas up to 15 terminal mismatches had little effect, strings of >5 mismatches distributed throughout a sequence, particularly mismatched G/C pairs, resulted in reduced signal; >12 mismatches distributed throughout a sequence resulted in no signal. On the basis of these findings, we pursued a conservative strategy in array design wherein a viral sequence was considered to be covered only if the array included at least 1 complementary probe with <5 mismatches.

The process for identifying bacterial, fungal, and parasitic probes was similar, although restricted to 16S and 18S rRNA sequences. Viral (GreeneChipVr) and panmicrobial (GreeneChipPm) array platforms were based on the GreeneVrdB and GreenePmdB, respectively. GreeneChipVr version 1.0 contained 9,477 probes to address all vertebrate viruses in the integrated ICTV/NCBI database (1,710 species, including all reported isolates) in 3 gene regions with <5 nucleotide mismatches. GreeneChipPm version 1.0 contained 29,495 probes that included probes comprising GreeneChipVr version 1.0, as well as 11,479 16S rRNA bacterial probes, 1,120 18S rRNA fungal probes, and 848 18S rRNA parasite probes. A total of 300 host immune response probes were added to arrays as a potential index to pathogenesis.

The 60-mer oligonucleotide arrays were synthesized on 70-mm × 20-mm glass slides by using an inkjet deposition system (Agilent Technologies, Palo Alto, CA, USA). A slide can accept up to 244,000 different 60-mer probes or 8 arrays, each comprising >15,000 probes. To facilitate alignment during scanning, 1,000 additional landing-light probes (5′-ATC ATC GTA GCT GGT CAG TGT ATC CTT TTT TTT TTA TCA TCG TAG CTG GTC AGT GTA TCC-3′) were placed in the corners and in a grid on the array. Fluorescently labeled synthetic oligonucleotides complementary to the control probes were included in all hybridizations.

### Viruses and Clinical Samples

Sources of viruses and viral reference strains used in this study are shown in Tables 1 and 2. Blood sample 200501379 (*Lake Victoria marburgvirus*, reference sample from Angola, 2005) and blood sample Angola-460 from a patient suspected of having viral hemorrhagic fever (VHF) were received in containers approved by the International Air Transport Association at either the Centers for Disease Control and Prevention in Atlanta, Georgia, USA or the Public Health Agency of Canada in Winnipeg, Ontario, Canada, respectively.

**Table 1:** DNA virus isolates from tissue culture samples used to test GreeneChip performance

Virus	Genus
Sealpoxvirus 1*	*Parapoxvirus*
Pseudocowpox virus†	*Parapoxvirus*
Orf virus†	*Parapoxvirus*
Cowpox virus†	*Orthopoxvirus*
Human herpesvirus 1*	*Simplexvirus*
Gallid herpesvirus 1†	*Iltovirus*
Human adenovirus E (HAdV-4)‡	*Mastadenovirus*
Human adenovirus C (HAdV-5)‡	*Mastadenovirus*

*University of Florida, Gainesville, FL, USA. †Commonwealth Scientific and Industrial Research Organization, Melbourne, Victoria, Australia. ‡American Type Culture Collection, Manassas, VA, USA.

**Table 2:** RNA virus isolates from tissue culture samples used to test GreeneChip performance

Virus	Genus
Negative-strand virus	
Lake Victoria marburgvirus†	*Marburgvirus*
Zaire ebolavirus‡	*Ebolavirus*
Sudan ebolavirus‡	*Ebolavirus*
Reston ebolavirus‡	*Ebolavirus*
Human respiratory syncytial virus A§	*Pneumovirus*
Human respiratory syncytial virus B§	*Pneumovirus*
Human parainfluenza virus 1§	*Respirovirus*
Human parainfluenza virus 3§	*Respirovirus*
Newcastle disease virus¶	*Avulavirus*
Vesicular stomatitis Indiana virus¶	*Vesiculovirus*
Bovine ephemeral fever virus¶	*Ephemerovirus*
Influenza A virus (H5N1)#	*Orthomyxovirus*
Influenza B virus§	*Orthomyxovirus*
Guanarito virus‡	*Arenavirus*
Machupo virus‡	*Arenavirus*
Junin virus‡	*Arenavirus*
Lassa virus strain Josiah‡	*Arenavirus*
Lassa virus strain Weller‡	*Arenavirus*
Positive-strand virus	
Human enterovirus B (E25)§	*Enterovirus*
Human enterovirus A (HEV71)§	*Enterovirus*
Human enterovirus B (E14)§	*Enterovirus*
Human enterovirus B (E30)§	*Enterovirus*
Vesicular exanthema of swine virus¶	*Vesivirus*
SARS* coronavirus**	*Coronavirus*
Human coronavirus OC43§	*Coronavirus*
Human coronavirus 229E§	*Coronavirus*
Dengue virus 1#	*Flavivirus*
Dengue virus 2#	*Flavivirus*
Dengue virus 3#	*Flavivirus*
Dengue virus 4#	*Flavivirus*
Yellow fever virus#	*Flavivirus*
West Nile virus**	*Flavivirus*
Saint Louis encephalitis virus**	*Flavivirus*
Alfuy virus††	*Flavivirus*
Murray Valley encephalitis virus††	*Flavivirus*
Chikungunya virus#	*Alphavirus*
Sindbis virus¶	*Alphavirus*
Double-strand virus	
Bluetongue virus¶	*Orbivirus*
Epizootic hemorrhagic disease virus-2¶	*Orbivirus*

*SARS, severe acute respiratory syndrome. †Centers for Disease Control and Prevention, Atlanta, GA, USA. ‡US Army Medical Research Institute of Infectious Diseases, Fort Detrick, Frederick, MD, USA. §American Type Culture Collection, Manassas, VA, USA. ¶Commonwealth Scientific and Industrial Research Organization, Melbourne, Victoria, Australia. #Centro Nacional de Microbiologica, Madrid, Spain. **Columbia University, New York, NY, USA. ††Curtin Institute of Technology, Perth, Western Australia, Australia.

Sources of clinical samples are shown in Table 3. Nasopharyngeal aspirates (SO4606 and SO5265) were collected by the Instituto de Salud Carlos III in Madrid, Spain, from children with respiratory disease. We also analyzed a nasopharyngeal aspirate (sample 23), a postmortem specimen from a patient who died of infection with severe acute respiratory syndrome coronavirus (SARS-CoV, sample TM-167), urine specimens from 2 patients with urinary tract infections (samples CUMC-NR7 and CUMC-NR9), a urine specimen from an asymptomatic patient (sample CUMC-LO1), and endometrial and lung tissues from a patient infected with *Mycobacterium tuberculosis* (samples CUMC-DL1 and CUMC-DL3).

**Table 3:** Clinical samples used to test GreeneChip performance

Pathogen	Genus	Sample
SARS* coronavirus	*Coronavirus*	Lung
Human respiratory syncytial virus A	*Pneumovirus*	Nasopharyngeal
Human enterovirus A (CAV10)	*Enterovirus*	Nasopharyngeal
Lake Victoria marburgvirus	*Marburgvirus*	Blood
Influenza A virus (H1N1)	*Orthomyxovirus*	Nasopharyngeal
*Klebsiella pneumoniae†*	*Klebsiella*	Urine
*Escherichia coli†*	*Escherichia*	Urine
*Mycobacterium tuberculosis‡*	*Mycobacterium*	Lung
*Mycobacterium tuberculosis‡*	*Mycobacterium*	Endometrial biopsy
*Lactobacillus* sp.§	*Lactobacillus*	Urine

*SARS, severe acute respiratory syndrome. †Detected on the array as a gammaproteobacterium. ‡Detected on the array as a mycobacterium. §Detected on the array as a lactobacillus.

### Sample Preparation and GreeneChip Hybridization

RNA was isolated from blood of VHF patients by using a 6100 Nucleic Acid PrepStation (Applied Biosystems, Foster City, CA, USA). RNA from virus isolates (culture supernatant) and other clinical samples (blood, nasopharyngeal aspirate, tissue, urine) was isolated by using the Tri-Reagent (Molecular Research Center Inc., Cincinnati, OH, USA). DNA was removed from RNA preparations by treatment with DNase I (DNA-free, Ambion Inc., Austin, TX, USA). First-strand reverse transcription was initiated with a random octamer linked to a specific primer sequence (5′-GTT TCC CAG TAG GTC TCN NNN NNN N-3′) (*5*). After digestion with RNase, cDNA was amplified by using a 1:9 mixture of the above primer and a primer targeting the specific primer sequence (5′-CGC CGT TTC CCA GTA GGT CTC-3′). Initial PCR amplification cycles were performed at a low annealing temperature (25°C); subsequent cycles used a stringent annealing temperature (55°C) to favor priming through the specific sequence. Products of this first PCR were then labeled in a subsequent PCR with the specific primer sequence linked to a capture sequence for 3 DNA dendrimers containing >300 fluorescent reporter molecules (Genisphere Inc., Hatfield, PA, USA), Products of the second PCR were added to sodium dodecyl sulfate–based hybridization buffer (Genisphere Inc.), heated for 10 min at 80°C, and added to GreeneChip for hybridization for 16 h at 65°C. After 10-min washes at room temperature with 6 × SSC (0.9 mol/L NaCl, 0.09 mol/L sodium citrate, pH 7.0), 0.005% Triton X-100, and 0.1 × SSC, 0.005% Triton X-100, Cy3 3DNA dendrimers were added and incubated at 65°C for 1 h. Slides were washed as before, air dried, and scanned (DNA Microarray scanner, Agilent Technologies).

### GreeneChip Analysis

Log-transformed analysis of microarrays using p values (GreeneLAMP) version 1.0 software was created to assess results of GreeneChip hybridizations. A map built from BLAST data was used to connect probe sequences to the respective entries in the GreenePmdB. Each of those sequences corresponds to an NCBI Taxonomy ID (TaxID). Individual TaxIDs were mapped to nodes in a taxonomic tree built based on ICTV virus taxonomy or the NCBI taxonomic classification for other organisms. The program output is a ranked list of candidate TaxIDs.

Probe intensities were corrected for background, log_2_-transformed, and converted to Z scores (and their corresponding p values). Where available, control-matched experiments from uninfected samples were used, and spots >2 standard deviations from the mean were subtracted. In instances where control-matched samples were not available, the background distribution of signal fluorescence in an array was calculated by using fluorescence associated with 1,000 random 60 mers (null probes). In both scenarios, positive events were selected by applying a false-positive rate of 0.01 (the rate at which null probes are scored as significant) and a minimum p value per probe of 0.1 in cases with a matching control and 0.023 (2 standard deviations) in cases without a matching control. Candidate TaxIDs were ranked by combining the p values for the positive probes for that TaxID by using the QFAST method of Bailey and Gribskov (*9*). This approach makes the following assumptions: 1) spot intensities are normally distributed; 2) spots represent independent observations (to minimize this effect clustering is used to collapse probes that are 95% identical); and 3) there are relatively few (<100) positive probes for any given TaxID. Probes for each kingdom (bacteria, eukaryotes, fungi, viruses) were analyzed independently to compensate for variations in signal-to-noise levels.

### Sequence Recovery from Hybridized Arrays

When a hybridization signal suggests a novel or chimeric agent, or the investigator wants to obtain sequence information, cDNA can be eluted for amplification and sequence analysis. A total of 100 μL of water at 90°C is added to the array and pipetted up and down 10 times. The eluate is recovered, amplified with the specific primer used during the initial amplification, and cloned into a plasmid vector (TOPO TA, Invitrogen, Carlsbad, CA, USA). After transformation into *Escherichia coli*, colonies are screened by sequencing. Primers based on the obtained sequence can be designed for confirmation of the agent or for specific (real-time) PCR screening of other specimens.

### Quantitative Real-Time PCR for *Plasmodium falciparum*

A quantitative real-time PCR assay was designed to amplify a 190-bp product from positions 178 to 367 of the 5.8S rRNA sequence eluted from the GreeneChipPm to confirm the presence of plasmodia in the original clinical sample. Reactions were performed in a 25-μL volume by using a commercial SYBR-Green reaction mixture (Applied Biosystems) and performed according to the manufacturer’s instructions. The primer sequences were 5′-GGAACGGCTTTGTAACTTGG-3′ and 5′-TGTCCTCAGAGCCAATCCTT-3′. The following cycling conditions were used: 50°C for 2 min and 95°C for 10 min, followed by 45 cycles at 95°C for 15 sec and 60°C for 1 min. To quantitate organism load in the original clinical sample, the targeted sequence region was cloned from the chip-hybridized, eluted nucleic acid. The cloned sequence was used to generate a 7-point standard curve (starting from 5 × 10^6^ copies/assay) for quantitation; each run included negative no-template controls. Thermal cycling was performed in an ABI 7300 real-time PCR system (Applied Biosystems).

## Results

### Evaluation of GreeneChip Performance

The performance of the GreeneChip system was initially tested in GreeneChipVr hybridizations that used extracts of cultured cells infected with adenoviruses, alphaviruses, arenaviruses, coronaviruses, enteroviruses, filoviruses, flaviviruses, herpesviruses, orthomyxoviruses, paramyxoviruses, poxviruses, reoviruses, and rhabdoviruses (49 viruses). All viruses were accurately identified (Tables 1 and 2). To assess sensitivity, viral RNA extracted from infected cell supernatants (adenovirus, West Nile virus, Saint Louis encephalitis virus, respiratory syncytial virus, enterovirus, SARS-CoV, and influenza virus) was quantitated by real-time PCR, serially diluted, and subjected to analysis with template concentrations ranging from 10 to 1,000,000 copies/assay. The threshold for detection of adenovirus (used as a DNA virus example) was 10,000 RNA copies; the threshold for detection of the RNA viruses tested was 1,900 RNA copies (Table 4).

**Table 4:** GreeneChip sensitivity for detection of various infectious agents*

Agent	Genus	Origin‡	Strain	Sensitivity
Human adenovirus E	*Mastadenovirus*	ATCC VR-1572	HAdV-4 RI-67	1.1 × 10^4^
Human adenovirus C	*Mastadenovirus*	ATCC VR-5	HAdV-5 Adenoid 75	3.2 × 10^4^
Human respiratory syncytial virus A	*Pneumovirus*	ATCC VR-26	Long	1.0 × 10^4^
West Nile virus	*Flavivirus*	GIDL	NY 99	1.9 × 10^3^
Saint Louis encephalitis virus	*Flavivirus*	GIDL	Parton	3.0 × 10^3^
SARS† coronavirus	*Coronavirus*	GIDL	Tor2	4.7 × 10^3^
Human enterovirus B	*Enterovirus*	ATCC VR-184	CBV4 strain JVB	5.2 × 10^3^
Influenza A virus H1N1	*Orthomyxovirus*	MSSM	A/New Caledonia/20/1999	9.8 × 10^3^

*Viral RNA extracted from infected cell supernatants was quantitated by real-time PCR, serially diluted, and subjected to GreeneChip analysis by using template concentrations ranging from 10^6^ to 10^1^ copies/assay. The threshold level of sensitivity for each virus tested is indicated. †SARS, severe acute respiratory syndrome. ‡ATTCC, American Type Culture Collection; GIDL, Jerome L. and Dawn Greene Infectious Disease Laboratory, Columbia University, New York, NY, USA.; MSSM, Mount Sinai School of Medicine, New York, NY, USA.

Array performance was then tested by using samples obtained from patients with respiratory disease, hemorrhagic fever, tuberculosis, and urinary tract infections. In all cases, array analysis detected an agent consistent with the diagnosis obtained by culture or PCR. GreeneLAMP analysis detected human enterovirus A, human respiratory syncytial A virus, influenza A virus, Lake Victoria Marburg virus (MARV), SARS-CoV, lactobacillus, mycobacteria, and gammaproteobacteria (Tables 1–3). Specific real-time PCR analyses indicated viral loads of 6.3 × 10^5^ copies/assay for SARS-CoV (*10*), 1.1 × 10^3^ copies/assay for respiratory syncytial virus (*11*), and 5.46 × 10^5^ copies/assay for enterovirus A (*12*) in clinical specimens. Details of the array analysis process are presented below for the detection of 2 viruses and 2 bacteria in clinical specimens.

**Figure 1:**
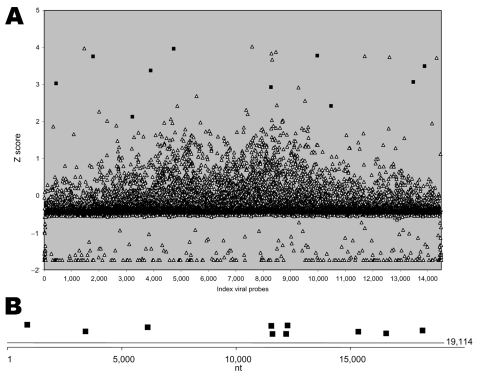
A) Signal intensity for viral probes in blood sample 200501379. Probe intensities were background corrected, log_2_-transformed, and converted to Z scores (and corresponding p values). Background distribution of signal fluorescence in the array was calculated by using fluorescence associated with 1,000 random null probes. Positive events were selected by applying a minimum p value per probe of 0.023 (2 standard deviations). Analysis of positive events with GreeneLAMP resulted in prediction of TaxID sample 11269 as the top prediction by the combined p value. Solid squares indicate Lake Victoria marburgvirus probes; open triangles indicate other probes. Ten of (90.9%) of 11 Lake Victoria marburgvirus probes were positive. B) Genomic location of positive Lake Victoria marburgvirus probes. Sequence-based analysis identified GenBank accession no. DQ447653 (Lake Victoria marburgvirus–Angola2005 strain Ang1379c) with 10 positive probes (all 8 motifs) as the best match.

Sample 200501379 contained RNA extracted from the blood of a person who died of VHF. In GreeneLAMP analysis, MARV TaxID 11269 was the top prediction by the combined p-value method using QFAST (*9*). The highest relative number of positive probes (10/11, 90.9%) also corresponded to MARV. In contrast, only 2 of 16 probes were positive for the next best predicted TaxID 11901, bovine leukemia virus (Figure 1A). Sequence-based analysis identified GenBank accession no. DQ447653 (Lake Victoria MARV–Angola2005 strain Ang1379c) with 8 positive probes as the best match. The 10 positive probes aligned with all 8 MARV gene motifs represented on the array (Figure 1B). Only 4 (17%) of 23 probes were positive for the next best predicted GenBank entry, AF534225 (*Gorilla gorilla* lymphocryptovirus 1); all aligned with only 1 motif.

Sample TM-167 contained RNA extracted from the lung of a person who died from SARS during the 2003 outbreak in Toronto, Ontario, Canada. In GreeneLAMP analysis, SARS-CoV was the top prediction by the combined p-value method. The highest relative number of positive probes (9/20, 45.0%) also corresponded to SARS-CoV. Sequence-based analysis identified GenBank accession no. AY274119 (SARS-CoV Tor2) with 9 probes representing 9 distinct genome motifs. The next best prediction was for AY738457 (influenza A virus); all influenza virus probes represented 1 genome motif.

Analyses of bacterial samples were more complex because many rRNA probes are cross-reactive between taxa, and the GreeneLAMP algorithm is not designed to take into account >100 probes positive for 1 TaxID. Thus, the program was run considering only probes that reacted with 1 genus-level TaxID. This strategy identified mycobacteria in sample CUMC-DL3 and lactobacilli in sample CUMC-LO1. In sample CUMC-DL3, the sequence-based algorithm identified AY725810 (uncultured *Mycobacterium* sp.) as significant, with 231 positive probes across 6 nonoverlapping regions. In sample CUMC-LO1, AJ853317 (*Lactobacillus vaginalis*) was the most significant result with 87 positive probes. Consensus PCR assays were developed for mycobacteria and lactobacilli. Primers designed by using Greene SCPrimer (http://scprimer.cpmc.columbia.edu/SCPrimerApp.cgi) were Myco_U901: 5′-ATCGAGGATGTCGAGTTGGC-3′ (forward); Myco_L968: 5′-TACTGGTAGAGGCGGCGATG-3′ (reverse); Lacto_817: 5′-CGGTGGAATGCGTAGATATATGGA-3′ (forward); and Lacto_1026: 5′-TCCTTTGAGTTTCAACCTTGCGGT-3′ (reverse). Products obtained after PCR amplification were sequenced and matched the predicted GenBank entries.

### Analysis of Unknown Sample from a Patient with VHF-like Syndrome

Within 6–8 days of infection, MARV causes an acute febrile illness that frequently progresses to liver failure, delirium, shock, and hemorrhage (*13*,*14*). From October 2004 through July 2005, a MARV outbreak in Angola resulted in 252 cases of hemorrhagic fever; 227 (90%) cases were fatal (*15*). Although most of the putative cases infected with MARV were confirmed by PCR, some were not. During this outbreak, a healthcare worker from a nongovernmental organization had acute fever and liver failure that culminated in death within 1 week. PCR assays of RNA extracted from blood showed no evidence of MARV infection. The same RNA was tested in a multiplex PCR for VHF that used primers for detection of Zaire Ebola, Sudan Ebola, MARV, Lassa fever, Rift Valley fever, Crimean-Congo hemorrhagic fever, Hantaan, Seoul, yellow fever, and Kyasanur Forest disease viruses (*3*) for differential diagnosis of VHF. Because this test did not identify an etiologic agent, the RNA was processed for panviral analysis with GreeneChipVr. Because no significant hybridization was detected, the RNA was assayed with GreeneChipPm. Bioinformatic analysis identified a *Plasmodium* sp. with 21 (62%) of 34 probes positive (Table 5). Chart review showed that the patient had recently arrived in Angola from a country where malaria was not endemic and that he had not taken malaria prophylaxis.

**Table 5:** Sequences of *Plasmodium*-reactive probes used to predict presence of plasmodia in blood sample Angola-460

Probe	Sequences (5′→3′)	Z score
Eu_5820_309	CGATTAATAGGAGTAGCTTGGGGGCATTTGTATTCAGATGTCAGAGGTGAAATTCTTAGA	3.699
Eu_5820_328	AGGGAGTGAAGACGCTCAGATACCGTCGTAATCTTAACCATAAACTATGCCGACTAGGCT	3.685
Eu_5820_322	ATAGGAGTAGCTTGGGGGCATTTGTATTCAGATGTCAGAGGTGAAATTCTTAGATTTTCT	3.681
Eu_5820_282	TTGTAATTGGAATGGTGGGAATTTAAAACCTTCCCAGAGTAACAATTGGAGGGCAAGTCT	3.672
Eu_5820_269	GCGTAAATTACCCAATTCTAAAGAAGAGAGGTAGTGACAAGAAATAACAATGCAAGGCCA	3.624
Eu_5820_296	TTAATAGGAGTAGCTTGGGGGCATTTGTATTCAGATGTCAGAGGTGAAATTCTTAGATTT	3.563
Eu_44417_518	ATCGTGATGGGGATAGATTATTGCAATTATTAATCTTCAACGAGGAATGCCTAGTAGGCG	3.558
Eu_5820_277	AACTGCGAAAGCATTTGCCTAAAATACTTCCATTAATCAAGAACGAAAGTTAAGGGAGTG	3.542
Eu_44417_516	GCATCGTGATGGGGATAGATTATTGCAATTATTAATCTTCAACGAGGAATGCCTAGTAGG	3.539
Eu_5820_325	CTTAGTTACGATTAATAGGAGTAGCTTGGGGGCATTTGTATTCAGATGTCAGAGGTGAAA	3.515
Eu_5820_298	GCAATTATTAATCTTGAACGAGGAATGCCTAGTAAGCATGATTCATCAGATTGTGCTGAC	3.507
Eu_5820_285	ATCGTCTTCACTCCCTTAACTTTCGTTCTTGATTAATGGAAGTATTTTAGGCAAATGCTT	3.432
Eu_5820_286	CTAACACAAGGAAGTTTAAGGCAACAACAGGTCTGTGATGTCCTTAGATGAACTAGGCTG	3.407
Eu_5820_311	GTCTAACACAAGGAAGTTTAAGGCAACAACAGGTCTGTGATGTCCTTAGATGAACTAGGC	3.347
Eu_5820_318	AATTATTAATCTTGAACGAGGAATGCCTAGTAGCATGATTCATCAGATTGTGCTGACTAC	3.290
Eu_5820_281	AAGTTTAAGGCAACAACAGGTCTGTGATGTCCTTAGATGAACTAGGCTGCACGCGTGCTA	3.282
Eu_5820_299	TCGATAACGAACGAGATCTTAACCTGCTAATTAGCGGTAAATACAACATATTCTTAAGTA	3.256
Eu_5820_308	TGATTGTAAAGCTTCTTAGAGGAACATTGTGTGTCTAACACAAGGAAGTTTAAGGCAACA	3.255
Eu_5820_324	AGTTTAAGGCAACAACAGGTCTGTGATGTCCTTAGATGAACTAGGCTGCACGCGTGCTAC	3.151
Eu_5820_275	TGATTGTAAAGCTTCTTAGAGGGACATTGTGTGTCTAACACAAGGAAGTTTAAGGCAACA	3.030
Eu_5820_301	CCCTGTTCTACTATAATTTGTTTTTTTTACTCTATTTCTCTCTTCTTTTAAGAATGTACT	2.834

**Figure 2:**
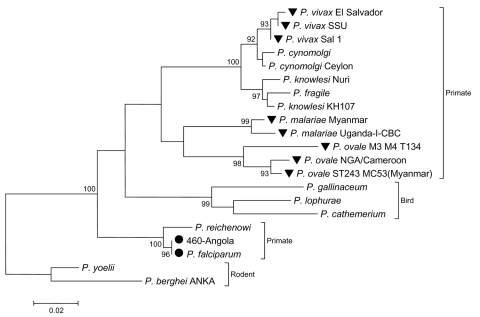
Analysis of 18S rRNA sequence (nt 291,256–292,364) recovered from the array after hybridization of sample Angola-460. The phylogenetic tree was reconstructed with the neighbor-joining method applying a Kimura 2-parameter model with MEGA version 3.1 (*16*). Number of nucleotide substitutions per site are indicated by the scale bar; bootstrap values (percentage of 1,000 pseudoreplicates) are given at relevant branches. Circles indicate *Plasmodium falciparum* sequences; inverted triangles indicate other known plasmodial pathogens of humans.

Hybridized cDNA was eluted from the array, cloned, and sequenced. Identified clones contained sequences corresponding to 18S rRNA and 5.8S rRNA of *P*. *falciparum* (Figure 2, Table 6). Plasmodia contain several alternative 18S-5.8S–28S rRNA genes. The expression of each rRNA set is developmentally regulated, which results in expression of a different set of rRNAs at different stages of the life cycle of the organism (*17*); e.g., S-type rRNA is expressed primarily in the mosquito vector, but A-type rRNA is expressed primarily in the human host (*17*). Only A-type sequences were recovered from the array. Analysis of the original RNA extract in a SYBR Green real-time PCR assay designed to amplify a 190-bp product of the *P*. *falciparum* 5.8S rRNA gene confirmed the presence of *P*. *falciparum* (2 × 10^6^ ± 8 × 10^4^ copies/µL blood), and indicated a parasite load >5%. The similarity of the signs and symptoms of severe malarial disease with viral hemorrhagic disease, the detection of a parasite load >5% (*18*), and the origin of this patient from a country nonendemic for malaria are consistent with a diagnosis of infection with *P*. *falciparum* as the most likely cause of death.

**Table 6:** Fragments of *Plasmodium falciparum* sequence recovered after GreeneChip hybridization of blood sample Angola-460

Clone	Position in the genome*	Size, nt	BLAST similarity
B06	286692–286986	295	100% *P*. *falciparum,* 98% *P*. *reichenowi*
D09	289685–289784	100	99% *P*. *falciparum,* 95% *P*. *berghei*
C01	291256–291624	369	100% *P*. *falciparum,* 98% *P*. *berghei*
A09	291521–291631	111	100% *P*. *falciparum,* 98% *P*. *berghei*
A08	291521–291614	94	100% *P*. *falciparum,* 98% *P*. *berghei*
H10	291521–291616	96	100% *P*. *falciparum,* 98% *P*. *berghei*
G02	291601–291637	37	100% *P*. *falciparum*
A01	291939–292088	150	100% *P*. *falciparum,* 98% *P*. *berghei*
J01	292039–292364	326	100% *P*. *falciparum,* 98% *P*. *berghei*

*Corresponds to GenBank accession no. AL929354 (*P*. *falciparum* strain 3D7, chromosome 5, segment 4/4, rRNA).

## Discussion

Differential diagnosis of hemorrhagic fevers poses challenges for clinical medicine and public health. Syndromes associated with agents are not distinctive, particularly early in the course of disease. In some instances, including the case presented here, >1 agent may be endemic in the region with an outbreak. Outbreaks caused by different agents may also overlap in time and geography. Examples of such coincident outbreaks include monkeypox and varicella-zoster viruses in the Democratic Republic of Congo in 1996 and 2001 (*19**,**20*) and measles and Ebola viruses in Sudan in 2004 (*21*). Furthermore, implicit in globalization is the risk of known or new agents that appear in novel contexts. In 1996, a presumptive diagnosis of Ebola VHF in 2 children who had recently returned to New York City from West Africa resulted in closing a hospital emergency room (*22*). One of the children died of cardiac failure caused by *P*. *falciparum* parasitemia and hemolysis (*23*). Therapeutic options for treatment of VHF are limited; however, rapid isolation of infected persons is critical to curb contagion. In contrast, whereas human-to-human transmission is not a primary concern with malaria, early specific therapy can have a profound effect on illness and death (*24*).

To address the challenges of emerging infectious diseases and biodefense, public health practitioners and diagnosticians need a comprehensive set of tools for pathogen surveillance and isolation. PCR methods have advantages with respect to sensitivity, throughput, and simplicity, but are limited in potential for multiplexing. Although microarrays have potential to allow highly multiplexed, unbiased surveillance, their use has been limited because of low sensitivity and unwieldy analytical programs. The GreeneChip system introduces sample preparation and labeling methods that enhance sensitivity, as well as user-friendly analytical software that we anticipate will facilitate clinical application. The advent of validated highly multiplexed microbiologic assays will afford unprecedented opportunities for unbiased pathogen surveillance and discovery and reduction of illness and death caused by infectious disease.
